# Park proximity, quality and recreational physical activity among mid-older aged adults: moderating effects of individual factors and area of residence

**DOI:** 10.1186/s12966-015-0205-5

**Published:** 2015-04-02

**Authors:** Jelle Van Cauwenberg, Ester Cerin, Anna Timperio, Jo Salmon, Benedicte Deforche, Jenny Veitch

**Affiliations:** Department of Public Health, Faculty of Medicine and Health Sciences, Ghent University, De Pintelaan 185, B-9000 Ghent, Belgium; Department of Human Biometry and Biomechanics, Faculty of Physical Education and Physical Therapy, Vrije Universiteit Brussel, Pleinlaan 2, B-1050 Brussels, Belgium; Fund for Scientific Research Flanders (FWO), Egmontstraat 5, B-1000 Brussels, Belgium; School of Exercise & Nutrition Sciences, Deakin University, Burwood Highway 221, Burwood, VIC 3125 Australia

**Keywords:** Retirement, Physical environment, Public open spaces, Leisure

## Abstract

**Background:**

The transition from active employment to retirement is a potentially critical period for promoting maintenance or development of recreational physical activity in older age. Park proximity and quality might be important correlates of recreational physical activity in this age group. However, research on park-physical activity relationships among mid-older aged adults is limited and inconclusive. Furthermore, while knowledge of individual moderators of park-physical activity relationships is crucial for tailoring interventions, this knowledge is also limited. We investigated relationships between perceived park proximity, park quality and recreational physical activity among mid-older aged adults. Additionally, we examined the potential moderating effects of gender, education level, retirement status, functional limitations and area of residence on these relationships.

**Methods:**

Self-reported data on demographics, functional limitations, park proximity, park quality, recreational walking and other recreational moderate- to vigorous-intensity physical activity (MVPA) were collected among 2700 Australian adults (57–67 years) in 2012. Objective information on area of residence was collected. To examine associations of park-related variables with recreational walking and other recreational MVPA, zero-inflated negative binomial (ZINB) regression models were used.

**Results:**

Park proximity significantly interacted with retirement status; non-retired participants who reported living near a park were more likely to participate in recreational walking, whereas no relationship was observed in retired participants. Among those who walked for recreation, higher park quality was related to more weekly minutes of recreational walking. No significant relationships with other recreational MVPA and no moderating effects of gender, education level, functional limitations and area of residence were observed.

**Conclusions:**

Parks may stimulate engagement in recreational walking among non-retirees and more walking among those who already walk. Future research should investigate which environmental factors relate to engagement in recreational walking among retirees and examine whether improvements in park quality actually lead to increases in mid-older aged adults’ recreational walking.

## Background

Worldwide, societies are ageing rapidly and face a burden of age-related diseases with significant economic and social costs [1]. The promotion of physical activity (PA) in the currently insufficiently-active population of mid-older aged adults is an effective strategy to prevent or delay the onset of age-related diseases and facilitate good health and independent living later in life [2,3]. A large proportion of mid-older aged adults in the Westernized world (55–69 years) experience a transitional phase in their lives (e.g., retirement and children leaving home) [4-8]. This transitional phase is a potentially critical period for ensuring maintenance or development of recreational PA, since daily routines and habits may be restructured and more leisure time may become available [9,10].

According to socio-ecological models, engagement in physical activity is determined by the interactions between a person’s individual characteristics and the environment in which he or she lives [11-13]. Therefore, the provision of attractive or high quality places for PA can be hypothesized to stimulate participation in recreational PA. Parks are a venue that might particularly stimulate recreational PA. Parks typically provide a green and restorative environment, can be used by whole communities at no/low cost (in comparison to gyms or sports clubs) and may include infrastructure for a variety of recreational physical activities (e.g. walking trails, sports courts) [14,15]. Indeed, higher levels of park use have been linked to higher levels of PA [14,16]. However, the relationship between park proximity and physical activity remains unclear. Several studies among adults and older adults reported the presence of nearby parks to be positively associated with different measures of physical activity [17-20], whereas other studies found no such relationship [21-24]. Similarly, measures of park quality have been linked to different measures of PA among adults and older adults in some studies [25-28], but not in others [24,29]. To our knowledge, no previous study has examined the relationships of park characteristics with PA specifically among mid-older aged adults.

A possible reason for the observed inconsistencies may be that park proximity and quality are not equally important for all individuals’ recreational PA. Relationships of park proximity and quality with recreational PA may be moderated by individual characteristics, such as gender, education level, retirement status, and functional limitations. While such interactions between individuals and their surrounding environments are at the core of socio-ecological models [11-13], this has rarely been studied, particularly among mid-older aged adults [16,30,31]. Neighborhood environment-physical activity relationships have been hypothesized to be stronger among older compared to younger adults. Due to retirement, older adults spend more time in their local neighborhood and, therefore, the neighborhood environment may exert a stronger influence on their recreational PA behaviors compared with their employed peers [32,33]. Hence, it could be hypothesized that the relationships of park proximity and quality with recreational PA are stronger among retired than among non-retired mid-older aged adults. Furthermore, press-competence models state that when functional competence decreases, the importance of a supportive neighborhood environment increases because it becomes more difficult to overcome environmental barriers (e.g. large distance to a park) [34]. Therefore, we hypothesized relationships of park proximity and quality with mid-older aged adults’ recreational PA to be stronger among those with more compared to less functional limitations. Knowledge of individual moderators is crucial for tailoring interventions to the needs of specific subgroups who are at increased risk of physical inactivity (e.g., women and the functionally limited) [35]. Additionally, the majority of previous research has been conducted in urban areas. Studies in rural areas are scarce [16,36,37], though park visitation and park features have been shown to differ between urban and rural areas [36,38]. Consequently, research is needed to examine whether relationships of park proximity and quality with PA are moderated by area of residence (urban vs. rural).

To summarize, evidence on the relationships between park proximity, quality and recreational PA is inconsistent and not specific to mid-older aged adults. Furthermore, there is a need to study the moderating effects of individual characteristics and area of residence on these relationships. Therefore, the current study aimed to investigate the relationships of perceived park proximity and park quality with recreational walking and ‘other’ moderate- to vigorous-intensity PA (MVPA) among a sample of Australian mid-older aged adults. Additionally, we examined the potential moderating effects of gender, education level, retirement status, functional limitations and area of residence (urban vs. rural) on these relationships.

## Methods

### Procedures and participants

The current study used data from the Wellbeing, Eating and Exercise for a Long Life (WELL) project, which has been described previously in full detail [39]. Briefly, the WELL study is a cohort study of mid-older adults aged 55–65 years at Time 1 (data collection in 2010) with follow-up at Time 2 (2012). The current study used cross-sectional data collected at Time 2 (measures of park proximity and quality were only introduced at Time 2). At Time 1, stratified cluster random sampling was used to recruit 55 to 65 year-olds residing in 84 suburbs that were randomly selected within urban and rural and low, medium, and high socio-economic strata within Victoria, Australia. Within each suburb, 134 participants (50% women) were randomly selected from the electoral roll (registration is compulsory in Australia) and invited to participate through postal mail. One week later, selected participants were sent a self-administered postal questionnaire with a reply-paid envelope. In total, 4,082 completed questionnaires were returned (38% response rate). Agreement to be re-contacted to participate in a follow-up questionnaire was obtained from 3368 participants (83%), of whom 2759 (82%) returned a questionnaire at Time 2. Participants who reported not being able to perform PA due to health restrictions and those who moved out of the State of Victoria between Time 1 and Time 2 were excluded, resulting in a final sample of 2700 participants. The Deakin University Human Research Ethics Committee approved the study protocol.

### Measures

#### Recreational walking and other recreational MVPA

Recreational walking and other recreational MVPA was assessed using the self-administered long version of the International Physical Activity Questionnaire (IPAQ-L), which has excellent test-retest reliability and acceptable validity in adults aged 15–69 years [40]. The IPAQ-L assessed the number of days in the last seven days during which participants walked for at least 10 minutes at a time for recreation. Consecutively, it assessed the average duration of walking on these days (hours and minutes/day). Frequency and duration were multiplied to obtain the variable ‘recreational walking’; expressed in minutes per week. Engagement in other recreational MVPA was assessed and calculated similarly. Participants were asked to indicate on how many days and for how long they were physically active at moderate (e.g. bicycling at a regular pace, swimming at a regular pace, and playing doubles tennis) and at vigorous intensity levels (e.g. aerobics, running, fast bicycling, or fast swimming) [40]. It is possible to undertake some of these other MVPA, such as running, cycling and some sports, in parks.

### Perceived park proximity and park quality

Perceived park proximity was assessed by asking the participants how long it would take them to walk from their home to the nearest park. This item was taken from the Neighborhood Environment Walkability Scale (NEWS) and standard scoring protocols were applied [41,42]. To assess satisfaction with park quality, participants indicated how much they agreed or disagreed with the statement ‘I am satisfied with the quality of parks in my neighbourhood’. Response options were a five-point Likert scale ranging from strongly disagree (1) to strongly agree (5). This variable was used as a proxy for perceived park quality.

### Individual factors

The following individual factors were assessed: date of birth, gender, education level, marital status, retirement status, years living at current address, and functional limitations. The physical functioning scale within the validated Short-Form 36-item Health Survey assessed functional limitations [43,44]. Participants indicated their level of limitation in performing ten activities of daily living (e.g., climbing several flights of stairs, vigorous activities such as running lifting heavy objects and participating in strenuous sports, lifting or carrying groceries, bending, kneeling or stooping, etc.) as: limited a lot (1), limited a little (1), or not limited (0).Activities in which participants reported to be limited a lot or a little were summed to create the variable ‘number of functional limitations’.

### Area-level variables

Suburbs were initially classified as urban or rural as reported by Cleland et al. [45]. Urban areas of Victoria included: (1) metropolitan Melbourne and (2) postcodes included within a 10 km radius of the centroid of regional cities (population >20,000). Areas outside metropolitan Melbourne and more than 25 km from the center of regional cities were classified as rural. Participants residing in a suburb in a ‘fringe’ local government area of Metropolitan Melbourne were excluded from the study at Time 1. Forty-five participants who moved to a fringe area between Times 1 and 2 were excluded from the moderation analysis of area of residence due to the small subsample size, but they were included in all remaining main effect and moderation analyses of this study.

The Socio-Economic Index For Areas (SEIFA), consisting of four measures of relative socio-economic (dis)advantage, economic resources, education and occupation, assigned by the Australian Bureau of Statistics [4] was used as an indicator for area-level socio-economic status (SES). Area-level SES was used as a covariate in all analyses.

### Statistical analyses

All analyses were conducted using Stata 10.1. To examine associations of park-related variables with the PA outcomes, zero-inflated negative binomial (ZINB) regression models with robust standard errors accounting for the hierarchical data structure (participants clustered within suburbs) were used. These models were used as the dependent variables were positively skewed and contained a large number of zero values. Vuong tests supported the need to use zero-inflated regression models [46]. ZINB models evaluate the relationships between the independent variables and the odds of non-participation in recreational walking/other recreational MVPA. Simultaneously, ZINB models estimate the relationships with weekly minutes of recreational walking/other recreational PA for participants who did engage in some recreational walking/other recreational PA. Hence, one ZINB model yields two regression coefficients for each independent variable: an odds ratio (OR) (for the relationship between the independent variable and the odds of not engaging in recreational walking/other recreational PA) and a negative-binomial model regression coefficient (representing the proportional change in minutes/week recreational walking or other recreational MVPA with a one-unit increase in the independent variable for participants engaging in recreational walking/other recreational PA).

First, a model that included the main effects of perceived park proximity, quality and all potential moderators was estimated. Second, five models were estimated which included the main effects and the interaction effect between the independent variables (perceived proximity and quality) and one of the five potential moderators. Third, a final model was built that combined the main effects with all significant moderators observed in the previous step. To keep the table simple and readable, we presented the results obtained in the first model in table and significant interaction effects observed in the final model were described in text. Significant interaction terms were probed according to established procedures [47]. Age and suburb-level socio-economic status were included as covariates in all analyses. All analyses with recreational walking as the dependent variable included other recreational MVPA as a covariate (and vice versa). Level of significance was determined at α = 0.05.

## Results

### Sample characteristics

Participants had a mean age of 62.3 (±3.1) years, 52.9% of the sample were women, 29.9% had obtained a university degree, 78.1% lived with a partner, 44.3% were retired, and 52.2% resided in a rural area (see Table 1). Participants reported a median of 15.0 years living at the current address and a mean of 2.7 (±2.6) functional limitations. The median value of walking for recreation was 70.0 minutes/week (mean ± standard deviation = 152.4 ± 212.3) and that of non-walking recreational MVPA was zero minutes/week (mean ± standard deviation = 97.1 ± 201.7).

**Table 1:** **Sample characteristics, perceived park proximity and quality and physical activity**

**Individual factors**	
Age (M ± SD, years)^a^	62.3 ± 3.1
Gender (% women)	52.9
Education level (%)	
<12 years of education	33.9
Year 12/trade/apprenticeship/certificate/diploma^a^	36.2
University degree	29.9
Marital status (%)	
Living with a partner	78.1
Separated/divorced/never married	16.9
Widowed	5.0
Retirement status (% retired)	44.3
Years living at current address (Mdn; Q1-Q3)^b^	15.0; 7.0 – 28.1
Functional limitations (M ± SD, number)	2.7 ± 2.6
**Area of residence (%)**	
Rural	52.2
Fringe	1.7
Urban	46.1
**Park characteristics**	
Perceived park proximity (M ± SD, /5)^c^	3.9 ± 1.3
Perceived park quality (M ± SD, /5)^d^	3.8 ± 0.9
**Dependent variables**	
Walking for recreation, mins/week (Mdn; Q1-Q3)	70.0; 0.0 – 210.0^e^
Other recreational PA, mins/week (Mdn; Q1-Q3)	0.0; 0.0 – 120.0^e^

M = Mean; SD = Standard Deviation; Mdn = Median; Q1 = quartile 1; Q3 = quartile 3.

^a^In Australia this refers to non-university tertiary education.

^b^For normally distributed continuous variables, means and standard deviations were presented. For non-normally distributed continuous variables, medians and interquartile ranges were presented.

^c^Perceived park proximity was assessed by asking the participants how long it would take them to walk from their home to the nearest park: 1–5 mins (5), 6–10 mins (4), 11–20 mins (3), 21–30 mins (2), 31+ mins (1), or don’t know (1). The option ‘don’t know’ was selected by 2.8% of the participants.

^d^Perceived park quality was measured on a five-point scale ranging from strongly disagree (1) to strongly agree (5) with the statement ‘I am satisfied with the quality of parks in my neighbourhood’.

^e^Walking for recreation, mins/week (M ± SD) = 152.4 ± 212.3.

Other recreational MVPA, mins/week (M ± SD) = 97.1 ± 201.7.

### Relationships with recreational walking

The logit model shows that perceived park proximity was significantly negatively related to the odds of non-participation in recreational walking (see Table 2). However, this relationship appeared to be significantly moderated by retirement status (OR interaction effect = 1.22; 95% CI = 1.05, 1.43; not shown in Table 2). In non-retired participants, a one-unit increase in park proximity was related to 14% lower odds of non-participation in recreational walking (OR = 0.86; 95% CI = 0.79, 0.94). In other words, non-retired participants who perceived that they lived near a park were more likely to participate in recreational walking. In contrast, park proximity was not significantly related to the odds of non-participation in recreational walking among retired participants (OR = 1.06; 95% CI = 0.95, 1.19). Park quality was not significantly related to the odds of non-participation in recreational walking.

**Table 2:** **Main effects of the potential moderators, perceived park proximity, and quality**

	**Recreational walking**	**Other recreational MVPA**		
	**Logit model: OR of being non-participant** ^**a**^ **(95% CI)**	**Negative binomial model: min/week (95% CI)**	**Logit model: OR of being non-participant** ^**b**^ **(95% CI)**	**Negative binomial model: min/week (95% CI)**
**Potential moderators**				
Gender (ref. = male)	0.72 (0.59, 0.88)**	0.99 (0.90, 1.90)	0.85 (0.70, 1.04)	0.85 (0.74, 0.99)*
Education level (ref. = ≤ 10 years of education)				
≤12 years of education/trade/certificate	0.86 (0.68, 1.99)	1.04 (0.93, 1.17)	0.80 (0.64, 0.99)*	1.00 (0.86, 1.17)
University degree	0.77 (0.61, 0.96)*	0.97 (0.97, 1.08)	0.47 (0.38, 0.61)***	0.88 (0.75, 1.04)
Retirement status (ref. = non-retired)	0.79 (0.63, 0.97)*	1.31 (1.19, 1.45)***	0.87 (0.69, 1.08)	1.18 (1.06, 1.32)**
Functional limitations	1.14 (1.09, 1.17)***	0.95 (0.93, 0.97)***	1.16 (1.12, 1.22)***	0.95 (0.92, 0.98)**
Area of residence (ref. = urban)				
Rural	0.82 (0.64, 1.05)	1.04 (0.95, 1.15)	1.09 (0.87, 1.38)	0.90 (0.77, 1.06)
Fringe	0.90 (0.43, 1.92)	0.84 (0.63, 1.11)	1.34 (0.70, 2.56)	0.77 (0.49, 1.20)
**Park characteristics**				
Park proximity	0.91 (0.85, 0.98)**	1.03 (0.99, 1.07)	0.98 (0.91, 1.05)	0.98 (0.93, 1.04)
Park quality	1.00 (0.98, 1.02)	1.02 (1.01, 1.02)***	0.94 (0.85, 1.03)	1.01 (0.94, 1.10)

OR = odds ratio; CI = confidence interval; *p < 0.05; **p < 0.01; ***p < 0.001.

^a^OR of being non-participant in recreational walking; ^b^OR of being non-participant in other recreational MVPA.

ZINB models evaluate two processes simultaneously. In the logit model, they analyze the relationships between the independent variables and the odds of non-participation in recreational walking or other recreational MVPA. In the negative binomial model, they analyze the relationships with weekly minutes of recreational walking or other recreational MVPA for participants who did engage in some recreational walking or other recreational MVPA. Negative binomial model parameters represent the proportional increase in minutes/week recreational walking or other recreational MVPA with a one-unit increase in the predictor.

The model for recreational walking included 2303 observations, of which 757 were zero observations, and was adjusted for age, suburb SES, and other recreational MVPA.

The model for other recreational MVPA included 2298 observations, of which 1406 were zero observations, and was adjusted for age, suburb SES, and recreational walking.

Regression coefficients in the negative binomial model showed that park proximity was not significantly related to weekly minutes of recreational walking. However, park quality was significantly positively related to weekly minutes of recreational walking with a one-unit increase in park quality related to 2% more weekly minutes of recreational walking. No significant moderating effects were observed in the negative binomial model.

### Relationships with other recreational MVPA

No significant relationships or moderating effects were found for park proximity and quality with other recreational MVPA in the logit or negative binomial part of the model (see Table 2).

## Discussion

The current study showed that self-reported perceptions of park proximity and quality were related to recreational walking but not to other recreational MVPA. Although other studies have shown park users to engage in moderate- and vigorous-intensity park-based activity [48,49], the current study showed that park proximity and quality might be important for stimulating recreational walking more than other forms of recreational PA among mid-older aged adults. This seems to suggest that other park-related variables not included in this study, such as the presence of specific features within parks (e.g., a sports facility), may be more important for higher intensity PA [17]. However, Schipperijn et al. [50] found that park features specifically aimed at PA did not relate to the use of a park for PA among 18- to 80-year-old Danish adults.

We found perceived park proximity and quality to be related to different levels of engagement in recreational walking. Proximity was related to engaging in any recreational walking, while park quality was related to the volume of recreational walking among those who walked for recreation. This supports findings in Australian (mean age 41 yrs.) [51] and Canadian (mean age 46 yrs.) adults [52], in which proximity-measures were related to engaging in any recreational walking or MVPA, whereas quality-measures (attractiveness and size) were related to achieving sufficient amounts of PA to obtain health benefits. Hence, having a nearby park might stimulate adults to walk to and in this park, but a high park quality might be necessary to encourage adults to spend time walking in the park and accumulate more minutes of walking. These findings are consistent with the the Transtheoretical Model, which hypothesizes that different determinants influence people’s health behaviours at different stages of change (e.g. contemplating about engaging in any recreational walking vs. already walking and maintaining this current level of recreational walking) [53]. Our findings indicate that the provision of a nearby park may stimulate older adults to start walking for recreation. On the other hand, increasing park quality may promote increased recreational walking in those who already walk for recreation. Not taking into account that environmental factors might relate differently to different “stages” of recreational walking might partially explain the inconsistent findings in previous studies [54]. More research is needed to disentangle how different characteristics of parks relate to different levels of engagement in recreational physical activity.

We observed an interaction effect between perceived park proximity and retirement status with higher park proximity related to an increased likelihood of engaging in any recreational walking, but only in those who were not retired. This contradicts our hypothesis that relationships would be stronger among retired older adults since they spend more time in their local neighborhood [32,33]. This unexpected finding may be explained by retirees having sufficient time available to travel to a park located further away, making park proximity less important. In contrast, due to time constraints non-retired adults might only engage in recreational walking if a park is located nearby their home. The concept of time budget is frequently used in transportation research, but to our knowledge has not been studied in the field of environment-PA relationships [55,56]. Alternatively, it might be that retirees have different activity patterns compared with non-retirees and, therefore, environmental characteristics other than park proximity (such as street design and traffic safety) may be more important to stimulate recreational walking among retirees. More research is needed to examine the influence of retirement on physical activity patterns and its moderating effect on environment-PA relationships.

A strength of the present study is its focus on mid-older aged adults, a relevant but understudied population who were living in urban and rural areas across suburbs with varying SES. Additionally, we examined possible moderating effects of several individual factors and area of residence. Knowledge of such moderating effects is limited despite their importance for effective intervention development [31]. The present study is limited by its exclusive reliance upon subjective rather than objective measures of park proximity and quality. Although, agreement between subjective and objective measures of park characteristics has been found to be low [57,58] both are hypothesized to have distinct influences on physical activity behaviors [59,60]. Schipperijn et al. [61] found subjective distance to the nearest park to be a better predictor of frequency of park use than objective distance. Future studies should include both subjective and objective measures of park characteristics to examine how they relate to recreational PA. Further, we assessed proximity and quality of the nearest park and it has been shown that the nearest park is not always the most frequently visited park [61]. Including measures of both the nearest and the most frequently visited park, would enable examination of which park characteristics are related to park visitation. In addition, perceived park quality was not assessed directly, but through a single-item question assessing satisfaction with park quality. Future studies may also benefit from the inclusion of a multi-item measure that includes different aspects of park quality (e.g. nuisance, presence of specific facilities etc.) to better understand which specific park characteristics are related to mid-older aged adults recreational PA. Furthermore, we assessed overall self-reported recreational walking and MVPA rather than park-based walking or MVPA which might have obscured the relationships with park proximity and quality. Combining Global Positioning System devices with accelerometry would yield objective and context-specific PA measures. While neighbourhood walkability (a composite index including residential density, street connectivity and land use mix diversity) has been linked previously to recreational walking among Belgian and US adults [62,63], it was not included in the WELL study. However, all current analyses were adjusted for ‘area of residence’, which might act as a proxy for neighborhood walkability since urban areas are typically denser and have a higher land use mix diversity than rural areas. Finally, the cross-sectional analyses do not allow us to infer causality; however, Kaczynski and Mowen [64] reported relationships between park availability and park-based PA to be preserved after accounting for residential self-selection. On the other hand, persons who are physically active may visit parks more often and, therefore, be more aware of park quality (i.e. poor park quality) than inactive persons who may visit parks less often. Longitudinal and experimental research is needed to establish causal relationships.

To conclude, non-retired mid-older aged adults who reported living near a park were more likely to engage in recreational walking compared to those living further away. No such relationship was observed among retirees. This seems to suggest that other environmental factors (e.g. street design and traffic safety) may be more important than park proximity in order to increase PA levels among retired persons. Better perceived park quality was related to the accumulation of more minutes of recreational walking among those who walked for recreation independently of retirement status, gender, education and functional level, and area of residence. The current findings imply that parks may stimulate engagement in recreational walking among non-retirees and more walking among those who already walk. Future research should investigate which environmental factors relate to engagement in recreational walking among retirees. Natural experiments are needed to examine whether improvements in park quality actually lead to increases in mid-older aged adults’ recreational walking.
